# Review of radiological classifications of pancreatic cancer with peripancreatic vessel invasion: are new grading criteria required?

**DOI:** 10.1186/s40644-017-0115-7

**Published:** 2017-05-06

**Authors:** Y. N. Shen, X. L. Bai, G. G. Li, T. B. Liang

**Affiliations:** 1 Department of Hepatobiliary and Pancreatic Surgery, Second Affiliated Hospital of Zhejiang University School of Medicine, Zhejiang University, Jiefang Road, Shangcheng District, Hangzhou, China; 2 Zhejiang Provincial Key Laboratory of Pancreatic Disease, Hangzhou, China

**Keywords:** Pancreatic cancer, Vessel invasion, Computed tomography criterion, Resectability, Review

## Abstract

Pancreatic cancer is mainly diagnosed at an advanced stage when adjacent vessel invasion is present; however, radical resection is potentially curative for selected patients with adjacent vessel invasion. Therefore, accurately judging the resectability of patients with adjacent vessel invasion represents a crucially important step in diagnosis and treatment. Currently, decisions regarding resectability are based on imaging studies, commonly contrast computed tomography (CT). Several radiological classifications have been published for vascular infiltration in pancreatic cancer. However, radiologists always formulate these CT grading systems according to their own experience, resulting in different judgment methods and parameters. And it is controversial in evaluating performance and clinical application. Besides, the conventional CT grading systems mainly focus on the evaluation of vessel invasion so as to less on the outcome of patient evaluation. In this review, we summarize the mainstream CT grading systems for vascular invasion in pancreatic cancer, with the aim of improving the clinical value of CT grading systems for predicting resectability and survival.

## Background

Pancreatic cancer is a highly lethal disease with high morbidity and a dismal prognosis [1, 2]. The 5-years survival rates for white and black American patients with pancreatic cancer are 8 and 7%, respectively, and the overall survival rate for all races is only 8% [2]. Approximately 90% of patients diagnosed with pancreatic cancer ultimately die of the disease [3]. Patients who do not have specific symptoms in the early stages are frequently diagnosed at an advanced stage, for which surgical therapy is usually not possible. Only 20% of patients with pancreatic cancer are eligible for one-stage resection [4]; however, 14 –30% of these cases will be found to be unsuitable for resection during surgery [5]. Therefore, the ability to accurately judge the resectability of pancreatic cancer represents a crucially important step in diagnosis and treatment, and could help to more accurately determine appropriate therapeutic approaches and predict the prognosis of individual patients. Moreover, once a patient is confirmed as unsuitable for surgery, palliative or neoadjuvant radiochemotherapy can be given in a timelier manner.

Computed tomography (CT) currently plays an important role in the diagnosis and stage evaluation of pancreatic cancer [6]. Preoperative CT evaluation of peripancreatic vascular infiltration in pancreatic cancer is an essential parameter used to assess whether resection can be performed. Several researchers [7–13] have assessed vascular involvement in pancreatic cancer and established a series of preoperative CT criteria to enable more accurate and reliable assessment. However, differences in imaging practices and interpretation [6], local experience and even the ethnicity of the patients have contributed to variations in these criteria, which are also limited by the technology and resources available. The clinical application of these criteria is also affected by their low accuracy. Therefore, it is imperative to establish widely-accepted criteria for vascular involvement in pancreatic cancer with higher precision and clinical value. Though the National Comprehensive Cancer Network (NCCN) established definitions for borderline resectable pancreatic cancer in 2014 [14] in which imaging features provide an important reference, we hold the opinion that the problems described above still persist. This review aimed to systematically summarize the mainstream CT criteria for peripancreatic vascular infiltration in pancreatic cancer published in recent few decades to provide a more comprehensive reference for radiologists and surgeons. Moreover, this information could contribute to the design and establishment of improved CT imaging criteria for vascular involvement in pancreatic cancer.

## Characteristics of existing criteria for vascular involvement in pancreatic cancer

### Loyer’s criteria (1996)

**Fig. 1:**
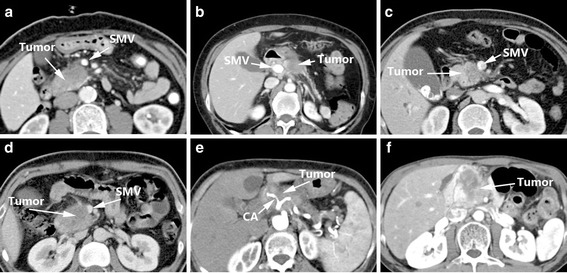
Loyer’s Criteria: Type A (**a**), Type B (**b**), Type C (**c**), Type D (**d**), Type E (**e**), Type F (**f**)

Loyer et al. [7] suggested CT criteria for vascular infiltration in pancreatic carcinoma in 1996 (Table 1). These criteria could be divided into six types (Type A – F) [7]: in Type A, a fat plane separates the tumor from adjacent vessels; Type B: normal pancreatic parenchyma separates the tumor from adjacent vessels; Type C: hypodense tumor not separated from vessels, and the points of contact form a convexity against the vessels; Type D: hypodense tumor not separated from vessels, and the points of contact form a concavity against or partially encircle the vessels; Type E: hypodense tumor encircled by adjacent vessels, while the fat plane between the tumor and blood vessels cannot be identified; and Type F: a tumor occluding the vessel (Fig. 1).

**Table 1:** Loyer’s Criteria [7]

Type	Imaging features
A	Fat plane separates tumor and/or normal pancreatic parenchyma from adjacent vessels.
B	Normal parenchyma separates hypodense tumor from adjacent vessels.
C	Hypodense tumor is inseparable from adjacent vessels, points of contact form a convexity against vessels.
D	Hypodense tumor is inseparable from adjacent vessels, points of contact form a concavity against or partially encircle vessels.
E	Hypodense tumor encircles adjacent vessels, no fat plane is identifiable between tumor and vessels.
F	Tumor occludes vessels.

For Type A/B pancreatic cancer, the resectable rate reached 100% (22/22). However, one patient with Type B accepted venous resection as normal pancreatic tissue was present within the tumor and around the portal vein, resulting in a resection rate without venous resection of 95% for Type A/B (21/22). For Type C, the resectable rate was 89% (8/9), and 55% for resection without venous resection. For Type D, the resectable rate was 47% (7/15), but only 7% for resection without venous resection (1/15).

Loyer’s criteria [7] were the first attempt to stratify patients with vascular invasion to distinguish clearly unresectable cases from potentially resectable cases [15]. The researchers calculated the resection rate for the included patients, which had a certain clinical significance. However, this method is complex and relatively subjective, and failed to provide definite definitions of resectable and unresectable tumors [15]. Moreover, Loyer et al. only paid attention to imaging features, and did not consider intra-operative and pathological findings, since a pathologist was not asked to prepare histologic sections of the vascular wall in the early cases [7]. This may have limited the accuracy of this system. In addition, arterial and venous infiltrations were not differentiated. There is another limitation as well: Type C is described as a hypodense tumor with a point of contact forming a convexity against the vessel. However, if a tumor, which is densely fibrotic, simply impinges the venous wall, thus having a convex border or point of contact with the vein, it would be classified as type C. However, this imaging finding was later-on called to be a “tear-drop deformity”, which is actually highly suggestive of venous wall invasion.

### Lu’s criteria (1997)

**Fig. 2:**
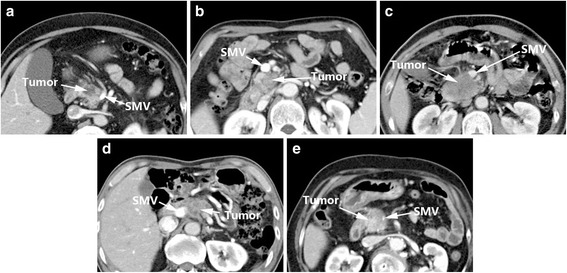
Lu’s Criteria: Grade 0 (**a**), Grade 1 (**b**), Grade 2 (**c**), Grade 3 (**d**), Grade 4 (**e**)

In 1997, Lu et al. [12] assessed 25 patients who underwent surgery for pancreatic adenocarcinoma and designed classification criteria for tumor resectability (Table 2). Imaging features of peripancreatic vessels were the main assessment for this criteria, and were classified into five grades: Grade 0: the tumor does not touch adjacent vessels; Grade 1: less than one quarter of the tumor circumference contacts vessels; Grade 2: one quarter to half of the tumor circumference contacts vessels; Grade 3: half to three quarters of the tumor circumference contacts vessels; Grade 4: over three quarters of the tumor circumference contacts vessels, or any vascular constriction (Fig. 2). When combined with intra-operative assessment, the higher the grade, the lower the resectability rate.

**Table 2:** Lu’s Criteria [12]

Grade	Imaging features
0	No contiguity of tumor to vessel.
1	Tumor contiguous to less than one-quarter circumference.
2	Between one-quarter and one-half circumference.
3	Between one-half and three-quarters circumference.
4	Greater than three-quarters circumferential involvement or any vessel constriction.

Lu’s criteria [12] considered a vessel circumferential involvement of 1/2 (180°) as the threshold of whether the tumor was resectable, which resulted in a sensitivity and specificity of 84 and 98%, respectively, and a positive predictive value (PPV) and negative predictive value (NPV) for unresectability of 95 and 93% (Table 7). These criteria were subsequently recognized and used by many scholars [16–18]. However, Lu’s criteria only focused on circumferential involvement, and ignored other important parameters like the length of tumor contact and stenosis, which could explain their relatively low sensitivity (84%). In addition, Valls et al. [15] stated that the main limitations of Lu’s criteria [12] were that only 11 patients were eventually resectable, and most of the surgical correlations were based on venous vessels.

### Li’s criteria (2005)

**Fig. 3:**
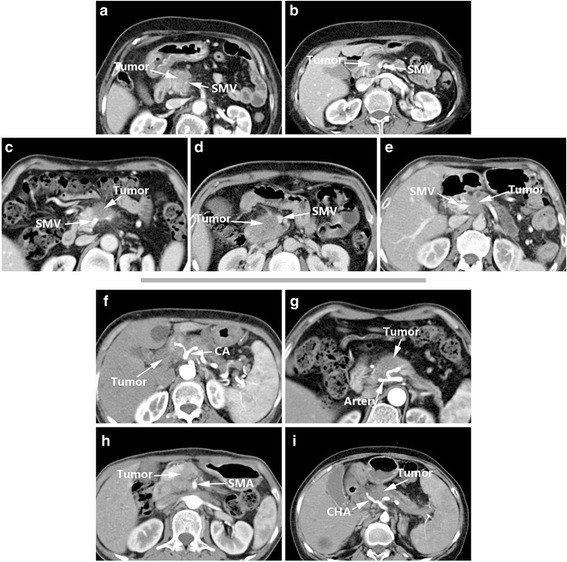
Li’s Criteria (vein): Sign A (**a**), Sign B (**b**), Sign C (**c**), Sign D (**d**), Sign E (**e**); Li’s Criteria (artery): Sign A (**f**), Sign B (**g**), Sign C (**h**), Sign D (**i**)

Li et al. reported sequential studies [8, 9] in 2005 and 2006 and designed a set of criteria for arterial and venous invasion in pancreatic cancer according to imaging features and intra-operative findings (Table 3). The criteria could be divided into four signs: Sign A: arteries embedded within the tumor or blocked veins; Sign B: circumferential involvement greater than 180°; Sign C: irregular vessel walls; and Sign D: vessel caliber stenosis. Then two criteria were recommended. Criteria of arterial invasion: presence of sign A, or combination of sign B with either sign C and/or D. Criteria of venous invasion: presence of one of the following signs: sign A, sign B, sign C, sign D and sign E (teardrop SMV) (Fig. 3).

**Table 3:** Li’s Criteria [8, 9]

Sign	Imaging features
A	Arterial embedment in tumor or venous obliteration.
B	Tumor surrounding 1/2 circumference of the vessel.
C	Vessel wall irregularity.
D	Vessel caliber stenosis.

Recommended criteria

Criteria of arterial invasion: presence of sign A, or combination of sign B with either sign C and/or D

Criteria of venous invasion: presence of one of the following signs: sign A, sign B, sign C, sign D and sign E (teardrop SMV)

The heterogeneity of Li’s criteria [8, 9] is acceptable, but this system had a low sensitivity when used for assessment of artery and venous involvement. Therefore, the researchers realized specific assessments are needed for arterial and venous invasion (Nakayama et al. [19] expressed a similar opinion in 2001). In their recommended criteria, Li et al. [8, 9] stated artery invasion may meet Sign A or Sign B combined with either Sign C or Sign D, and venous invasion may meet Sign A, Sign B, Sign C, Sign D or Sign E (teardrop shape performance of the superior mesenteric vein). The sensitivity of these arterial and venous assessments for vessel invasion reached 79% (23/29) and 92% (45/49), respectively (Table 7). The researchers considered that venous and arterial invasion present different CT signs of invasion, because the venous wall is thinner and weaker than the muscular arterial wall. When veins are surrounded or infiltrated by tumor, the wall tends to be irregular and the calibre becomes narrowed. At the same time, tumor often penetrates the venous wall and forms thrombus since the flow rate in veins becomes slow, causing venous occlusion finally [8].

### Klauss’s criteria (2008)

**Fig. 4:**
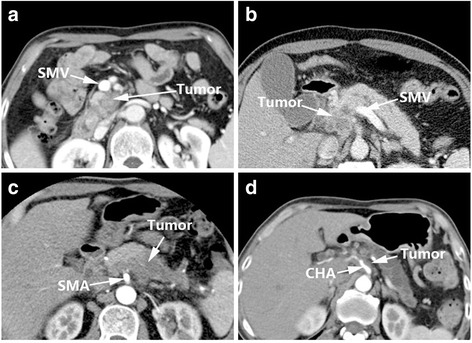
Klauss’s Criteria (vein): Score < 11 (**a**), Score > 11 (**b**); Klauss’s Criteria (artery): Score < 11 (**c**), Score > 11 (**d**)

In 2008, Klauss et al. [13] proposed a new preoperative CT system to assess the resectability of pancreatic cancer (Table 4) based on the relation of peripancreatic vessels to the tumor, and verified the results using intra-operative findings and postoperative pathological reports. In this system, artery and venous assessments are separate (Fig. 4). Compared to the previous versions described above, Klauss’s criteria include more assessment items and more detail. For example, the venous assessment includes assessment of the length of tumor contact, circumferential involvement and other abnormalities; the length of tumor contact and circumferential involvement are recorded to an accuracy of mm and degrees. The length of tumor contact and circumferential involvement assessment was also added for the artery assessment. Furthermore, this system provides a corresponding score for each assessment item, and the total score is calculated by adding the score for each item after the assessment. Generally, the total score was used to judge the resectability of the tumor and assess peripancreatic vessel invasion. Finally, 11 points was selected as the cut-off point for evidence of vessel invasion. That is to say, the vessel was invaded by the tumor in case of the total score of single vessel was equivalent with or higher than 11 points.

**Table 4:** Klauss’s Criteria [13]

Length of tumor contact (mm)	Circumferential Involvement (°)	Other abnormalities	Score
Veins			
0	0		1
< 5	1–45		2
5–10	46–90		3
11–20	91–180	Flattened	4
21–40	181–270	Long-segment contour deformity	5
> 40	> 270	Obliteration or severe contour deformity	6
Total score			∑
Arteries			
0	No		1
< 5	In Places		2
5–10	Continuously < 45		3
11–20	45–180		4
21–40	181–270		5
> 40	270 to complete obliteration		6
Total score			∑

One major limitation of Klauss’ Score is the fact that this very meticulous scoring system was developed with the same patient cohort, which was then also used to validate the score. For such an advanced scoring system a separate validation cohort would have been reliable. Based on their criteria, Klauss et al. [13] verified whether the superior mesenteric vein (SMV), superior mesenteric artery (SMA), splenic vein and portal vein (PV) or celiac trunks were involved in each patient. The sensitivity of this method for vessel invasion reached 66.7 to 100% (Table 7). Among the 28 patients, the sensitivity and specificity of the tumor resectability assessment reached 95.5% (21/22) and 100% (6/6), respectively [13]. Compared to other related systems or criteria, Klauss’s criteria have a higher sensitivity and specificity and warrant increased use in the clinic. However, Klauss et al. [13] stated their criteria also had a number of limitations, including the fact benign tumors would also lead to vessel compression and could lead to diagnostic errors.

### Marinelli’s criteria (2014)

**Fig. 5:**
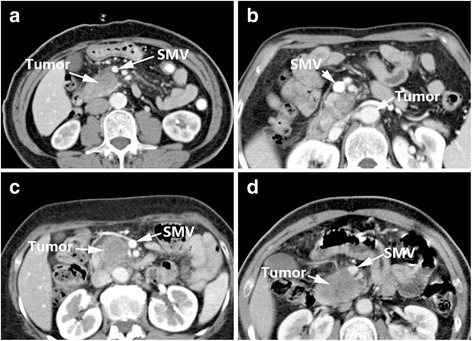
Marinelli’s Criteria: Grade 1 (**a**), Grade 2 (**b**), Grade 3 (**c**), Grade 4 (**d**)

The assessment system designed by Marinelli et al. [10] (Table 5) was mainly designed to assess peripancreatic venous invasion such as portal vein (PV) and superior mesenteric vein (SMV), with the aim of selecting the appropriate therapeutic approach after accurate preoperative assessment to improve the treatment and prognosis of patients with borderline resectable disease. Compared to other criteria, the design of this system is more complicated. The items assessed are: tumor contact with vessel, length of tumor contact, circumferential involvement and stenosis. It is noteworthy that the tumor contact with vessel criterion employed the system included in Loyer’s criteria [7]. However, interestingly Marinelli et al. [10] combined Loyer’s Grade A and Grade B in their system. Marinelli et al. maintained there is no significant difference between these two grades in terms of surgical outcome, since both are clearly resectable [10]. The “length of tumor contact” was classified as 0 mm, < 5 mm and > 5 mm; “circumferential involvement” as 0°, 0° to 90°, 90° to 180°, and > 180°, respectively. Four grades were defined in Marinelli’s criteria [10]: Grade 1, definite absence of invasion; Grade 2, probable absence of invasion; Grade 3, probable presence of invasion; and Grade 4, definite presence of invasion. In Grade 1, the tumor contacts with vessels of Grade A–B, and length of tumor contact is 0 mm with circumferential involvement of 0 and no stenosis. In Grade 2, the tumor contacts with vessels of Grade C, with a length of tumor contact < 5 mm, circumferential involvement is 0°–90° and no stenosis. There are two kinds of situations in Grade 3, the tumor contacts with vessels of Grade C, with a length of tumor contact > 5 mm, circumferential involvement is 0°–90° or flattened vessels. Another situation is circumferential involvement is 0–90° with flattened vessels, Grade D tumor vessel contact. Grade 4 includes three scenarios: grade E or F tumor vessel contact and circumferential involvement > 180°; narrowing of vessels; or Grade D tumor contact, contact length > 5 mm and circumferential involvement of 90° to 180° (Fig. 5).

**Table 5:** Marinelli’s Criteria [10]

Grade (likelihood of vascular invasion)	Tumor contact with vessel^a^	Length of tumor contact with vessel (mm)	Circumferential vein involvement (°)	Stenosis
1	Grade A–B	= 0 mm	= 0°	No stenosis
2	Grade C	< 5 mm	= 0°–90°	No stenosis
3	Grade C–D	> 5 mm	= 0°–90°	Flattened
4	Grade D	> 5 mm	>90° < 180° >180°	Occlusion thrombus
	Grade E/F	-		-

Grade 1, Definite absence of invasion; Grade 2, Probable absence of invasion; Grade 3, Probable presence of invasion; Grade 4, Definite presence of invasion

^a^Grades A–F, according to Loyer’s Criteria [8]:

Grade A: fat plane visible between tumour and vessels

Grade B: normal pancreatic tissue between tumour and vessels

Grade C: tumour adjacent to vessel with a convex contour towards vessels

Grade D: tumour adjacent to vessel with a concave contour towards vessels

Grade E: circumferential involvement of vessels

Grade F: vascular occlusion

The advantage of Marinelli’s score over Klauss’ criteria [13] is the fact that Marinelli’s scoring system refers to actual clinical situations instead of adding score numbers. Marinelli et al. [10] verified their standard in 56 patients with pancreatic cancer and obtained sensitivity and specificity values for PV invasion of 80 and 100%, respectively. The PPV and NPV were 80 and 96%. For the SMV, the sensitivity and specificity of this method reached 100 and 94%, and the PPV and NPV were 75 and 100% (Table 7). The innovation in this method was that the researchers analyzed the prognosis of the patients by grade. For the PV infiltration score, the survival time was inversely proportional to grade, though the trend was not significant (*P* = 0.106). Additionally, the researchers proposed that both the PV and SMV infiltration scores were associated with metastatic disease and the resection margins status [10].

### Teramura’s criteria (2016)

**Fig. 6:**
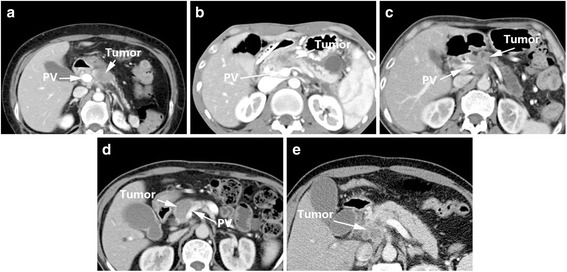
Teramura’s Criteria: Type 0 (**a**), Type 1 (**b**), Type 2 (**c**), Type 3 (**d**), Type 4 (**e**)

Teramura et al. [11] assessed whether pathological PV invasion (pPV) in pancreatic cancer could be accurately identified by preoperative CT in order to select patients who could benefit from surgery. The researchers established a CT diagnostic standard according to the degree of vascular invasion, intra-operative findings and pathology results (Table 6). The classification method for this criteria is similar to Loyer’s criteria [7] and is divided into five types (Type 0 – 4): In Type 0, a fat plane separates the tumor and (or) normal pancreatic tissues from adjacent vessels; in Type 1, soft tissue density exists between the tumor and vessels; in Type 2, the tumor cannot be separated from the adjacent vessels and the points of contact from a convexity against the vessels; in Type 3, the PV is deformed, narrowed or exhibits stenosis; and in Type 4, the PV is completely blocked by the tumor (Fig. 6).

**Table 6:** Teramura’s Criteria [11]

Type	Diagnosis	CT findings	
0	Negative	Negative	c vessels.
1		Soft tissue density	Soft tissue density between tumor and portal vein.
2	Positive	Contact	Tumor is inseparable from adjacent vessels, and points of contact from a convexity against the vessels.
3		Stenosis	Deformation, narrowing or stenosis on portal vein.
4		Obstruction	Portal vein is completely obstructed by tumor.

Teramura et al. [11] demonstrated that the prognosis of Type 0 vs. Type 3/4 was significantly different (*P* = 0.02), but not for Types 0 vs. 1/2 and Types 1/2 vs. 3/4 (*P* = 0.30 and *P* = 0.10, respectively). The 5-years survival rates for Type 0, 1/2 and 3/4 were 23.1, 11.4 and 3.2%, respectively. Although a significant difference in 5-years survival was not observed between Type 1/2 and Type 3/4, a higher percentage of patients with Type 1/2 than Type 3/4 survived for 36 months (10/35 vs. 1/32). Therefore, this method was feasible to assess whether patients are suitable for pancreaticoduodenectomy with PV resection via preoperative CT [11]. However, while the sensitivity and NPV were 97.6 and 97.5%, respectively, the specificity was only 60% and the PPV was 61.2% (Table 7).

**Table 7:** Sensitivity and specificity of each criteria for vessel invasion in pancreatic cancer

Criteria	Vessel	Sensitivity (%)	Specificity (%)	PPV (%)	NPV (%)
Loyer et al. (*n* = 56)	NA	NA	NA	NA	NA
Lu et al. (*n* = 25)	Vein/Artery	84	98	95	93
Li et al. (*n* = 54)	Vein	92	100	NA	NA
	Artery	79	99	NA	NA
Klauss et al. (*n* = 28)	SMV	100	95.8	80	100
	Splenic vein	66.7	100	100	96.2
	PV	100	96.2	66.7	100
	Celiac trunk	100	100	100	96.4
	SMA	100	100	100	96.4
Marinelli et al. (*n* = 56)	PV	80	100	80	96
	SMV	96	94	75	100
Teramura et al. (*n* = 107)	PV/SMV	97.6	60	61.2	97.5

*PV* portal vein, *SMV* superior mesenteric vein, *SMA* superior mesenteric artery, *NA* not available, *PPV* positive predictive value, *NPV* negative predictive value

## Clinical significance

With respect to resectability, patients with Type A and B vascular involvement according to Loyer’s criteria [7] are suggested to undergo pancreatic resection, while Type E and F are considered inoperable. In their study, one case of Type E/F underwent surgery with vessel resection, though a positive margin was detected in the pathological examination. Resection was recommended for Type C, but the tumor may or may not attach to the vessel wall. A detailed plan of the surgical approach should be made before pancreatic resection in cases of Type D. It is important to note that venous resection should not be attempted if the surgeon lacks relevant experience. In addition, Loyer’s [7] study did not provide a definite definition of resectable and unresectable, as previously discussed. Teramura et al. [11] mainly focused on the relationship between prognosis and vascular invasion. They reported patients with “stenosis”, “obstruction”, or a Klauss score [13] ≥ 11 are likely to have a poor prognosis, even with portal vein reconstruction (PVR) [11], and recommended resectability should be assessed from the perspective of prognosis. According to the aforementioned data, Lu et al. [12] used one-half of the circumference of the vessel as the threshold; resection should be recommended if the value was higher. Furthermore, Li et al. [8, 9] and Hough et al. [20] found that a tear drop appearance of the SMV can be a contraindication for resection. However, unambiguous definitions of resectable tumors were not provided in the criteria by Klauss [13] and Marinelli [10].

In recent years, these rigid concepts of vascular invasion (meaning non-resectability) have been somehow overruled by the concept of “borderline resectable”, which has been adopted by many cancer centers and institutions. According to the NCCN guidelines (Version 1.2017), the “borderline resectable” could be defined as several resectability statuses as follows:

1. Venous
  Solid tumor contact with SMV or PV of > 180°, contact of < = 180° with contour irregularity of the vein or thrombosis of the vein but with suitable vessel proximal and distal to the site of involvement allowing for safe and complete resection and vein reconstruction;
  Solid tumor contact with the inferior vena cava (IVC).
2. Arterial
  2.1 Pancreatic head/uncinate process:
    Solid tumor contact with common hepatic artery (CHA) without extension to celiac axis or hepatic artery bifurcation allowing for safe and complete resection and reconstruction;
    Solid tumor contact with the superior mesenteric artery (SMA) of < = 180°;
    Solid tumor contact with variant arterial anatomy (ex: accessory right hepatic artery, replaced right hepatic artery, replaced CHA, and the origin of replaced or accessory artery) and the presence and degree of tumor contact should be noted if present as it may affect surgical planning.
  2.2 Pancreatic body/tail:
    Solid tumor contact with the celiac axis (CA) of < = 180°;
    Solid tumor contact with the CA of > 180°without involvement of the aorta and with intact and uninvolved gastroduodenal artery thereby permitting a modified Appleby procedure.

Interestingly, Teramura et al. [11] doubted the definition of “borderline” pancreatic head cancer established in the newest NCCN guidelines, and pointed out that circumferential contact of the PV did not have high diagnostic value and may even affect assessment of the resectability of “borderline” pancreatic head cancer.

Other studies also considered prognosis. Nakao et al. [21] showed the imaging features of PV correlated with long-term survival; survival was poorer for patients with bilateral narrowing or stenosis/obstruction with collaterals than patients with unilateral narrowing [21]. Moreover, they also suggested that radiographic classification of PV invasion was more appropriate than pathological classification [21]. A similar report by Chun et al. [22] showed patients with bilateral narrowing were less likely to benefit from preoperative treatment.

Another useful feature of these criteria [7–13] summarized by us is the prediction of vascular invasion. Some researchers previously believed perivascular changes were not specific for pancreatic carcinoma [23–25]. However, Megibow maintained that patients with pathological confirmed ductal adenocarcinoma are likely to have tumor infiltration if perivascular changes can be observed on CT [26]; this supposition was supported by Loyer [7]. In the study by Zeman et al. [27], vascular invasion could be identified if the caliber was irregular, circumferential involvement > 180°, or vessel thrombosis was present. In the study by Furukawa et al. [28], vascular invasion is classified as positive if circumferential involvement is more than 90°. Klauss et al. [13] considered it was difficult to assess vascular invasion as the contact between the vessels and tumor does not always indicate whether the vessels have been truly infiltrated; Teramura et al. [11] expressed a similar view. In research published in 2012, Nakao et al. [21] found imaging classifications of PV invasion correlated with the pathological grade of invasion.

## Conclusions

From the information above, we can conclude that the previous studies suggesting criteria for assessing the resectability of pancreatic cancer via CT are basically consistent, and while some criteria are suitable for clinical practice (for example, the sensitivity and specificity of the Klauss’s criteria [13] reach 95.5 and 100%, respectively), they also remain controversial. In most studies, the length of tumor contact, circumferential involvement, stenosis and other imaging findings are taken as reference items. However, in the latest study, Teramura et al. [11] reported circumferential involvement had low diagnostic value and they removed this feature from their criteria. In addition, scoring systems with a high reference value like Klauss’s criteria also have limitations. For instance, it is difficult to distinguish whether vessels are oppressed by a benign or malignant tumor on CT.

In conclusion, we hold the opinion that the current criteria [7–13] have superior clinical value to previous systems, The scoring system, especially from Klauss’ and Marinelli’s, is worthy of being applied to the clinical practice. However, the criteria above still remain controversial, especially with respect to the lack of the prognostic criteria. We believe that with continuous developments in CT technology and accumulation of experience by radiologists, more improved and accurate criteria will be established.

## Abbreviations

CA: Celiac axis
CHA: Hepatic artery
CT: Computed tomography
NCCN: National comprehensive cancer network
NPV: Negative predictive value
PPV: Positive predictive value
PV: Portal vein
SMA: Superior mesenteric artery
SMV: Superior mesenteric vein
